# Association between Japanese Diet Adherence and Muscle Weakness in Japanese Adults Aged ≥50 Years: Findings from the JSTAR Cohort Study

**DOI:** 10.3390/ijerph20227065

**Published:** 2023-11-15

**Authors:** Akio Shimizu, Kiwako Okada, Yasutake Tomata, Chiharu Uno, Fumiya Kawase, Ryo Momosaki

**Affiliations:** 1 Department of Food and Health Science, Faculty of Health and Human Development, The University of Nagano, 8-49-7, Miwa, Nagano 380-8525, Japan; 2 Institute of Health and Nutrition, Nagoya University of Arts and Sciences, 57, Iwasaki-cho, Nisshin 470-0196, Japan; kiwako@nuas.ac.jp (K.O.); chiharu83724@gmail.com (C.U.); 3 School of Nutrition and Dietetics, Faculty of Health and Social Services, Kanagawa University of Human Services, 1-10-1, Heisei-cho, Yokosuka 238-8522, Japan; toomata-5h0@kuhs.ac.jp; 4 Graduate School of Nutritional Science, Nagoya University of Arts and Sciences, 57, Iwasaki-cho, Nisshin 470-0196, Japan; 21gn101@st.nuas.ac.jp; 5 Department of Rehabilitation Medicine, Mie University Graduate School of Medicine, 2-174, Edobashi, Tsu 514-8507, Japan; momosakiryo@gmail.com

**Keywords:** diet, epidemiology, geriatrics, sarcopenia, frailty

## Abstract

Japanese diet adherence has been inversely correlated with muscle weakness. In this study, we aimed to validate that association. Longitudinal data from 1699 individuals aged ≥50 years (mean age 62.5 ± 6.9 years, 50.4% female) at two time points (2007 and 2011) were used. Participants without muscle weakness from several regions in Japan were included. The 12-component revised Japanese Diet Index (rJDI12) classified by tertiles assessed adherence to the Japanese dietary pattern. Muscle weakness was defined as a handgrip strength of ˂18 kg for females and ˂28 kg for males based on the Asian Working Group for Sarcopenia criteria 2019. A multivariate logistic approach was used to determine the relationship between rJDI12 tertile and the occurrence of muscle weakness by calculating the odds ratio (OR) and its 95% confidence interval (95% CI) throughout the observation period. Muscle weakness was negatively correlated with the highest rJDI12 tertile (OR [95% CI] 0.891 [0.814, 0.973] for T3). This association was consistent in sensitivity analyses with multiple imputations of missing values. Closely following the Japanese dietary pattern appears to reduce the occurrence of muscle weakness among the aging population in this study, suggesting it may prevent frailty and sarcopenia in the aging population.

## 1. Introduction

Handgrip strength is an indicator of overall muscle power and a crucial biomarker [1] for diagnosing conditions like dynapenia [2], sarcopenia [3,4,5], and physical frailty [6]. Several studies and meta-analyses have shown that muscle weakness, defined by low handgrip strength, is linked to increased mortality risk in the middle-aged and older population, irrespective of race [1,7,8,9]. Moreover, muscle weakness is correlated with adverse events such as falls and fractures [1] and worse instrumental activities of daily living (IADL) [10]. Muscle strength begins to decline in middle age [11]. Protein intake from middle age and older is considered important to maintain muscle health [12]. In addition, previous study has reported that exercise habits in middle age are inversely associated with the prevalence of sarcopenia in old age [13]. Therefore, it is vital to make healthy lifestyle choices, including exercise and diet, to maintain muscle strength from an early stage.

Healthy dietary habits may play a vital role in maintaining muscle strength or preventing muscle weakness in the aging population. Previous studies have shown that higher intakes of beneficial nutrients such as vitamin C, polyunsaturated fatty acids, and dietary fiber are associated with higher handgrip strength [14]. Additionally, antioxidants like vitamins C and E, along with polyunsaturated fatty acids, are essential for skeletal muscle synthesis and proper functioning [15,16]. Consistent adherence to healthy diets, such as the Japanese and Mediterranean diets, has been shown to be negatively correlated with the prevalence of muscle weakness [17,18]. It has been postulated that healthy dietary patterns contribute to health through the synergistic effects of various nutrients and foods rather than through the intake of individual nutrients [19]. Consequently, adherence to healthy dietary patterns may be more effective for maintaining muscle strength or preventing muscle weakness.

The Japanese dietary pattern, characterized by a higher intake of soy products, fish, vegetables, rice, seaweed, miso soup, pickles, and green tea, has numerous health benefits [20]. Decreased risk of functional disability [21] and mortality [22] have been associated with adherence to this dietary pattern. A previous study has demonstrated an association between Japanese dietary patterns and adequate nutrient intake [23]. Furthermore, a cross-sectional study found that adherence to the Japanese diet correlated with lower rates of muscle weakness in the middle-aged and older Japanese population [17]. In light of these findings, and considering muscle weakness as a cause of physical disability [24], adherence to the Japanese dietary pattern may be effective not only in reducing physical disability and mortality but also in preventing muscle weakness.

However, to our knowledge, this association has not been studied longitudinally. A causal relationship between Japanese dietary patterns and muscle strength could be established by conducting a longitudinal study. This longitudinal study aimed to investigate the association between adherence to the Japanese dietary pattern and the occurrence of muscle weakness, as measured by handgrip strength.

## 2. Materials and Methods

### 2.1. Study Design

We conducted a longitudinal study using data from the Japan Study of Aging and Retirement (JSTAR) cohort survey. The JSTAR database has been described in detail elsewhere [17,25]. Briefly, this panel survey targeted Japanese individuals aged ≥50 years living in 10 cities across Japan and was conducted in four waves (2007, 2009, 2011, and 2013). The JSTAR dataset was compiled through the collaborative efforts of the Research Institute of Economy, Trade and Industry, Hitotsubashi University, and the University of Tokyo as part of the Survey of Life and Health. Survey items encompassed current food consumption, medical history, education, smoking, alcohol consumption, weight, height, handgrip strength, IADL, and daily walking time.

**Figure 1:**
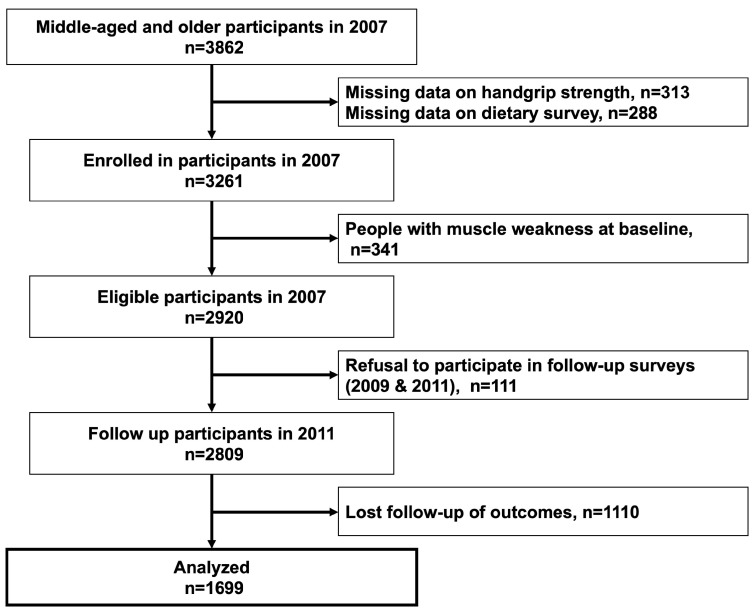
Study participant selection pathway.

To minimize sampling bias, data from 2007 and 2011, with the most prolonged follow-up periods and the fewest lost follow-ups, were used. The baseline survey was carried out from 17 January to 16 July 2007, whereas the follow-up survey occurred from 17 May 2011 to 21 February 2012. This survey involved a mailed questionnaire for participants (self-completion or “drop off” questionnaire) and computer-assisted interviews (computer-assisted personal interviews). Figure 1 illustrates a flowchart of survey participation.

### 2.2. Ethics and Informed Consent

In the JSTAR survey, informed consent was obtained from all participants at the time of recruitment. This research protocol was approved by the Ethics Committee of the Nagoya University of Arts and Sciences (approval number: 578).

### 2.3. Measurements

#### 2.3.1. Dietary Survey Questionnaire

Dietary intake data were gathered using the brief-type self-administered diet history questionnaire (BDHQ). This questionnaire was designed to obtain information on nutrient and food intakes from regularly consumed foods (excluding dietary or similar supplements). The questions evaluated intake during the previous month based on the food preferences of adults living in Japan [26,27]. The survey contains 80 items and estimates the consumption of 58 types of foods and more than 100 different nutrients. Standard meal portions at BDHQ have been researched and determined from several Japanese recipe books. In addition, the amount of energy and protein obtained from the BDHQ was also categorized in terms of gender-based quartile.

#### 2.3.2. Evaluation of Japanese Dietary Pattern

We used the 12-component Revised Japanese Dietary Index (rJDI12), established in a previous study to assess adherence to the Japanese dietary pattern [17,28]. The rJDI12 is based on the previously created JDI [20], modified to a healthier dietary pattern more suited to the dietary patterns of the modern Japanese population [28]. The rJDI12 includes beneficial items such as rice, miso, fish, shellfish, green and yellow vegetables, seaweed, pickles, green tea, coffee, soybeans, soy-derived foods, fruit, and mushrooms. Conversely, beef and pork are included as less beneficial items [17,28]. For beneficial components, 1 point was added if intake exceeded the gender-based median, whereas for less beneficial components, 1 point was added if intake was less than the gender-based median. As a result, the rJDI12 score ranged from 0 to 12, with higher scores indicating a higher adherence to Japanese dietary patterns. Previous studies have demonstrated an inverse association between rJDI12 scores and both cognitive decline [28] and muscle weakness [17].

#### 2.3.3. Calculating the Overall Nutrient Adequacy Score

To investigate the relationship between consistency with dietary patterns and nutrient sufficiency, we employed the Overall Nutrient Adequacy (ONA) score [23]. A detailed description of the ONA score calculation can be found elsewhere [23]. In brief, the ONA score is computed based on nutrient adequacy ratios for nine items (protein, fiber, vitamins A, C, E, potassium, calcium, iron, and magnesium) and two items that should be limited (saturated fat and sodium). Nutrient adequacy ratios were determined according to the recommended dietary allowance values of the Dietary Reference Intakes for Japanese, and each nutrient, excluding saturated fatty acids, was standardized by gender-stratified energy intake. In this study, the ONA score was calculated based on the nutrient intake obtained from the BDHQ.

#### 2.3.4. Assessment of Muscle Strength

During each survey session, we used a Smedley-style handheld dynamometer (No. 6103, TANITA, Tokyo, Japan) to measure the handgrip strength (in kilograms) of the predominant hand by instructing to grip the dynamometer as firmly as possible [25]. Handgrip strength is strongly correlated with lower limb muscle strength, which reflects whole-body muscle strength [29]. Therefore, its use as an indicator of whole-body muscle strength has been reported to be acceptable in older adults [29].

#### 2.3.5. Covariates

Covariates included gender, age category, body mass index (BMI) category, IADL, self-reported comorbidities, drinking and smoking status, education level, and time spent walking per day. Age categories were 50–54 years, 55–59 years, 60–64 years, 65–69 years, and 70 years and above. BMI (kg/m^2^) was calculated from self-reported height (m) and body weight (kg). BMI was categorized according to value into <18.5 kg/m^2^, 18.5–25 kg/m^2^, ≥25 kg/m^2^, or missing categories. IADL was assessed using the Tokyo Metropolitan Institute of Gerontology Index of Competence (TMIG-IC) [30]. There are five items in the TMIG-IC: (1) use of public transportation (buses and trains), (2) shopping for daily necessities, (3) meal preparation, (4) bill payment, and (5) bank use. Disability of IADL is defined as a deficiency in the ability to perform any one of the five TMIG-IC items [30]. Based on the disability of IADL, the participants were categorized as yes, no, or missing. Self-reported comorbidities included cerebrovascular diseases, coronary heart diseases, diabetes, and cancer. Self-reported comorbidities were categorized as yes, no, or missing for each item. Drinking status was categorized as every day, sometimes, never, or missing. Smoking status was defined as current smoker, former smoker, never smoked, or missing. Education levels were categorized as ≥16 years, <16 years, or missing. They were categorized according to their time spent walking per day as ≥1 h, ≥0.5–<1 h, <0.5 h/day, or unknown.

#### 2.3.6. Outcome

The occurrence of muscle weakness at follow-up in 2011 was the primary outcome of this study. Muscle weakness was identified as a grip strength of <18 kg for females and <28 kg for males based on the Asian Working Group for Sarcopenia criteria (AWGS) 2019 [4]. Muscle weakness based on the AWGS 2019 definition has been reported to be a predictor of mortality in older hospitalized patients [31].

### 2.4. Statistical Analysis

We presented categorical variables as numerical values (percentages) and other variables as medians (interquartile range). The chi-square test was applied to categorical variables, and the Kruskal–Wallis test to other variables. We used Spearman’s rank correlation coefficient to assess the correlation between adherence to the Japanese dietary pattern and the ONA score. We employed multivariate-adjusted logistic regression models to calculate odds ratios (ORs) and 95% confidence intervals (CIs) for muscle weakness. Participants were categorized into tertiles based on their rJDI12 scores, representing adherence to the Japanese dietary pattern, ranging from lowest to highest adherence. Dummy variables were created for these categories, with T1 (low) serving as the reference category. The rJDI12 score was also incorporated as a continuous variable in each model.

Our models adjusted for several factors. In Model 1, we adjusted for gender, age category (50–54 years, 55–59 years, 60–64 years, 65–69 years, or 70 years and above), and baseline handgrip strength. To examine whether the rJDI12 association with muscle weakness was due to body size effects, Model 2 added BMI (<18.5 kg/m^2^, 18.5–25 kg/m^2^, ≥25 kg/m^2^, or missing). To further explore the impact of physical health status and lifestyle, Model 3 incorporated Model 2 adjustments along with disability of IADL (no, yes, missing), time spent walking (≥1 h/d, 0.5–1 h/d, <0.5 h/d, or missing data), drinking status (every day, sometimes, never, or missing data), smoking status (current, former, never, or missing data), comorbidities (cerebrovascular disease, coronary heart disease, diabetes, cancer [yes, no, or missing data for each term]), and educational level (<16, ≥16 y, or missing), with corrections for these factors. Lastly, to investigate the effects of energy and protein intake, Models 4 and 5 were adjusted by adding energy intake (kcal/d; gender-based tertile categories) and protein intake (g/d; gender-based tertile categories) to Model 3, respectively.

To evaluate the competing risk of death in relation to muscle weakness, we conducted an additional analysis on the association between adherence to the Japanese dietary pattern and the composite outcome (muscle weakness and death) [32].

We assumed that the missing information was missing at random (MAR). To impute missing or unevenly distributed data to the MAR, multiple imputation was performed using the Multiple Imputation by Chained Equations (MICE) package [33] in R, excluding participants who were missing handgrip strength at baseline. In the sensitivity analysis, we created 50 data sets with multiple imputed data and pooled the estimates for each data set in a multivariate logistic regression model to obtain odds ratios adjusted for covariates. All data were analyzed using R version 4.2.3 (R Core Team, Vienna, Austria). All statistical tests were two-sided, with *p* < 0.05 considered significant.

## 3. Results

Our study cohort initially included 3862 participants from the first wave in five cities (Adachi, Kanazawa, Shirakawa, Sendai, and Takikawa) who provided valid responses. We excluded 313 participants with missing initial handgrip strength data from 2007 and 288 participants with incomplete dietary survey data. Additionally, 341 participants with muscle weakness at baseline, 111 participants who refused to participate in follow-up surveys, and 1110 participants with unavailable outcome data after four years were also excluded from the analysis. Consequently, we analyzed data from a final sample of 1699 participants. 

The demographic details of the participants, categorized by the tertiles of rJDI12 scores, are outlined in Table 1. Among this population, 49.6% were male, with a mean age of 62.5 ± 6.9 y and a mean BMI of 23.4 ± 3.0 kg/m^2^. The mean follow-up period for these participants was 4.4 ± 0.2 years. Participants with higher rJDI12 scores were older (*p* < 0.001), had lower BMIs (*p* = 0.013), were less likely to be current smokers (*p* = 0.004), and walked more than one hour (*p* = 0.022). Moreover, higher rJDI12 scores were higher in energy (*p* < 0.001) and protein (*p* < 0.001) intake.

**Figure 2:**
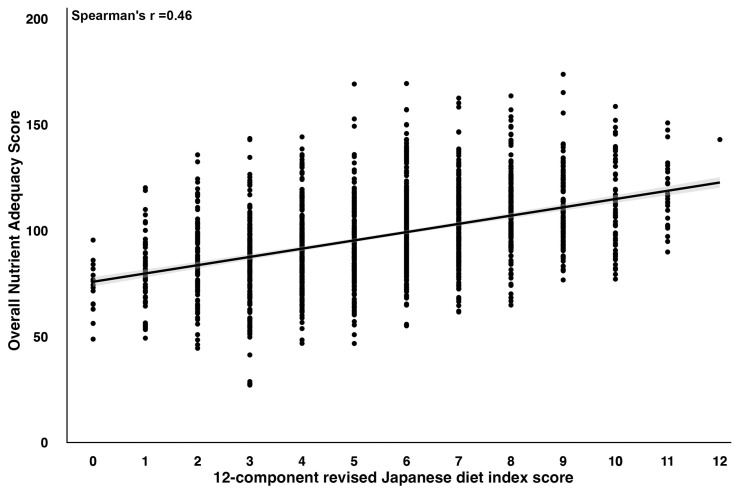
Correlation between the rJDI12 scores and overall nutrient intake.

No notable variance in initial handgrip strength was observed across the tertiles of rJDI12 scores (*p* = 0.261). Energy and protein intakes, which affect muscle strength, were higher among participants with higher rJDI12 scores. A moderate correlation was observed between the ONA score and rJDI12 score (Spearman’s r = 0.46), indicating that higher rJDI12 scores correlated with overall nutrient adequacy (Figure 2).

**Figure 3:**
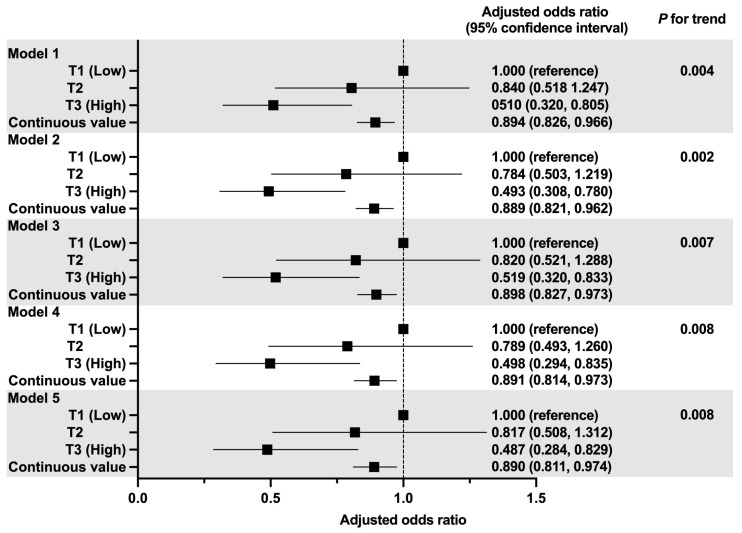
Relationship between the rJDI12 scores and occurrence of muscle weakness. Model 1 adjusted for gender, age category, and handgrip strength at baseline. Model 2 adjusted for model 1 and added the BMI category. Model 3 was adjusted for model 2 and added disability of IADL, drinking status, smoking status, disease history, educational levels, and time spent walking. Model 4 was adjusted for Model 3 and added energy intake. Model 5 adjusted for Model 3 and added protein intake. Continuous values were entered into each model for rJDI12.

The correlation between the rJDI12 score and the occurrence of muscle weakness is illustrated in Figure 3. In the age- and sex-adjusted model (Model 1), higher tertiles of the rJDI12 score were inversely associated with muscle weakness (ORs [95% CIs] 0.510 [0.320, 0.805] for T3).

Similar results were obtained for Model 2, adjusted for BMI, which affects muscle strength (ORs [95% CIs] 0.493 [0.308, 0.780] for T3). Similar results were observed in Model 3 adjusted for lifestyle (ORs [95% CIs] 0.519 [0.320, 0.833] for T3). Higher tertiles of the rJDI12 score were inversely associated with the occurrence of muscle weakness, even after adjusting for energy and protein intake affecting muscle strength (OR [95% CI] 0.498 [0.294, 0.835] for T3 in Model 4, and OR [95% CI] 0.487 [0.284, 0.829] for T3 in Model 5). This association remained consistent when the rJDI12 score was treated as a continuous variable (ORs [95% CIs] 0.891 [0.814, 0.973] in model 4). 

Supplementary Figure S1 presents the sensitivity analysis results using MICE to account for missing information. In age- and sex-adjusted model (Model 1), higher tertiles of the rJDI12 score were inversely associated with muscle weakness (ORs [95% CIs] 0.510 [0.320, 0.805] for T3). Similar results were observed in Model 2, adjusted for BMI, which affects muscle strength (ORs [95% CIs] 0.537 [0.336, 0.858] for T3). Similar results were observed in Model 3 adjusted for lifestyle (ORs [95% CIs] 0.544 [0.338, 0.874] for T3). Higher tertiles of the rJDI12 score were inversely associated with the occurrence of muscle weakness, even after adjusting for energy and protein intake affecting muscle strength (OR [95% CI] 0.556 [0.333, 0.928] for T3 in model4, and OR [95% CI] 0.548 [0.325, 0.923] for T3 in model 5). In addition, point estimates and 95% CIs were similar for all models.

Table 2 presents a sensitivity analysis examining the association between the rJDI12 tertile score and the composite outcome (muscle weakness or death), considering competing risks of death. The ORs for the composite outcome tended to be lower with higher tertiles of the rJDI12 score (OR for T3 [95% CI] 0.636 [0.402, 1.002]). However, no such association was observed for the occurrence of death alone (ORs [95% CIs] 1.479 [0.616, 3.709] for T3).

## 4. Discussion

In this longitudinal study, we evaluated the association between adherence to the Japanese dietary pattern and the occurrence of muscle weakness among middle-aged and older adults using a nationwide database in Japan. Notably, the reduced occurrence of muscle weakness correlated with higher adherence to the Japanese dietary pattern, and this finding was robust even after accounting for missing information using MICE.

The observation period of the present study was approximately 4 years. In a previous study in community-dwelling older Chinese adults, with a follow-up period of 4 years, the occurrence of sarcopenia was identified [34]. Considering this finding, the observation period in this study was considered long enough to identify the occurrence of muscle weakness.

Higher adherence to the Japanese dietary pattern correlated with a reduced occurrence of muscle weakness. This finding was consistent with cross-sectional results in which adherence to healthy dietary patterns was inversely correlated with muscle weakness [17,18,35]. Furthermore, adherence to the Japanese dietary pattern has been reported to be associated with a lower prevalence of sarcopenia, characterized by muscle weakness and low muscle mass [36]. The Japanese dietary pattern is characterized by a high intake of fish, soy products, vegetables, and seaweed, as well as a moderate intake of rice while being low in saturated fats and refined sugars [23]. These components provide an array of essential nutrients, such as animal and vegetable proteins, dietary fiber, and various vitamins and minerals, which may contribute to improved muscle health. A systematic review has indicated that omega-3 fatty acids found in fish enhance muscle strength among older adults [37]. In this study, adherence to the Japanese dietary pattern was also inversely correlated with the occurrence of muscle weakness in a multivariate analysis that adjusted for energy and protein intake, which both affect muscle strength. Additionally, soy protein, prominent in the Japanese dietary pattern, has been shown to be more effective than whey protein in enhancing muscle strength [38]. Furthermore, adherence to the Japanese dietary pattern correlated with overall nutrient satisfaction. Adequate nutrient intake is important for maintaining muscle strength [39]. A systematic review also reported that nutrient sufficiency is positively correlated with sarcopenia-related parameters such as muscle strength and muscle mass [40]. Therefore, the Japanese dietary pattern adherence might contribute to handgrip strength maintenance through better nutrient intake. 

The possibility that the dietary patterns of the Japanese may influence the maintenance of muscle strength may be explained by differences from other dietary patterns. For instance, the Japanese diet is known for its rich content of omega-3 fatty acids from fish consumption, which have been shown to promote muscle health and function by reducing inflammation and improving muscle protein synthesis [16]. In contrast, Western diets tend to have higher levels of omega-6 fatty acids, which may promote inflammation [41] and have less beneficial effects on muscle health. Additionally, the Japanese dietary pattern emphasizes plant-based foods such as soy products, which may have a positive impact on overall health and muscle strength due to their bioactive components, including isoflavones and essential amino acids like leucine. This differs from other healthy dietary patterns, which place less emphasis on soy products and their corresponding nutrients. Thus, the combination of nutrients and food groups emphasized in the Japanese dietary pattern, such as omega-3 fatty acids, soy products, and plant-based foods, may help explain its potential benefits for muscle health.

In our sensitivity analysis considering adherence to the Japanese dietary pattern and death, we did not observe a statistically significant association. In the short term, from 2007 to 2011, various factors other than dietary patterns may have played a more substantial role in mortality, such as pre-existing medical conditions, accidents, or other lifestyle factors like smoking and alcohol consumption. Previous studies that investigated the effect of Japanese dietary patterns on mortality had an observation period of approximately 10 years [22,42]. The relatively short duration of the study may not have allowed for a clear assessment of the long-term impact of the Japanese dietary pattern on mortality risk.

The findings of this study have the potential to inform a population-based approach to preventing frailty and sarcopenia, both of which are geriatric syndromes. Notably, dietary patterns represent modifiable aspects of one’s lifestyle. There is growing evidence to suggest that healthy dietary patterns play a crucial role in delaying the aging process [43]. Hence, it is imperative to advocate for healthy dietary patterns as a means to foster healthy aging.

The strength of this study is that it included older adults living in multiple regions of Japan. Thus, the findings of this study may be generalizable to the Japanese population. However, this study has several limitations. First, it could not adjust for all potential confounding factors. Muscle weakness is influenced by lifestyle factors such as exercise, and although daily walking time was considered in this study, there may be other residual confounding factors. Second, potential bias may have existed because many cases that were lost to follow-up were excluded from the analysis. This could have led to an underestimation or overestimation of the true association between the Japanese dietary pattern and muscle weakness if the excluded participants had different dietary patterns or risk factors affecting their muscle health. However, the results were considered robust because they were consistent even when missing information was substituted using MICE [33], a statistical method that helps to reduce bias in the findings. Finally, the dietary pattern analyzed in this study utilized the BDHQ, a food intake frequency survey method tailored to Japanese portion sizes. Given that the food intake frequency results from the BDHQ can be prone to errors, employing the dietary weighing method would have been preferable. Nonetheless, many epidemiological studies employ food intake frequency survey methods, including the Food Frequency Questionnaire and BDHQ.

## 5. Conclusions

Our study suggests that adherence to the Japanese dietary pattern protects muscle strength among middle-aged and older Japanese adults. Further research is needed to explore the potential benefits of the Japanese dietary pattern in other populations and age groups.

## Figures and Tables

**Table 1:** Characteristics of the study participants by adherence to the Japanese diet.

Characteristics	Japanese Diet Index Tertile			
	T1 (Low)	T2	T3 (High)	*p* Value
Range of scores	0–4	5–6	7–12	
Participants, n	518	565	616	
Age, y, median (IQR)	60.0 (56.0–67.0)	62.0 (57.0–68.0)	64.0 (58.0–69.2)	<0.001
Female, n (%)	264 (51.0)	273 (48.3)	319 (51.8)	0.468
Body mass index, kg/m^2^, median (IQR)	23.5 (21.5–25.5)	23.2 (21.5–25.4)	22.9 (21.1–24.7)	0.013
History of disease, n (%)				
- Cerebrovascular diseases	14 (2.7)	16 (2.8)	12 (1.9)	0.498
- Coronary heart diseases	49 (9.5)	59 (10.4)	63 (10.2)	0.706
- Diabetes	51 (9.8)	45 (8.0)	53 (8.6)	0.465
- Cancer	17 (3.3)	22 (3.9)	20 (3.2)	0.646
Current smoker, n (%)	134 (25.9)	121 (21.4)	100 (16.2)	0.004
Current drinker, n (%)	301 (58.1)	357 (63.2)	379 (61.5)	0.156
Educational level ≥ 16 y, n (%)	149 (28.8)	140 (24.8)	150 (24.4)	0.141
Time spent walking ≥ 1 h/d, n (%)	200 (38.6)	241 (42.7)	302 (49.0)	0.022
Disability of instrumental activities of daily living, n (%)	19 (3.7)	23 (4.1)	24 (3.9)	0.943
Handgrip strength, kg, median (IQR)	29.0 (24.0–36.0)	30.0 (24.0–38.0)	30.0 (23.0–38.0)	0.261
Energy, kcal/d, median (IQR)	1566 (1267–1866)	1935 (1630–2300)	2296 (1957–2742)	<0.001
Protein, g/d, median (IQR)	55.5 (44.3–66.7)	74.3 (59.6–87.5)	91.9 (76.2–112.3)	<0.001

**Table 2:** Relationship between tertiles of the rJDI12 score and the composite outcome to consider competing risk of death.

	Total, n	Event	Odds Ratio (95% Confidence Interval)	*p* Value
		n (%)		
Occurrence of muscle weakness or death (composite outcome)				
T1 (low)	518	67 (12.9)	1.000 (reference)	
T2	565	63 (11.2)	0.810 (0.531–1.232)	0.324
T3 (High)	616	69 (9.7)	0.636 (0.402–1.002)	0.051
Death				
T1 (low)	518	10 (1.9)	1.000 (reference)	
T2	565	14 (2.5)	1.072 (0.452–2.621)	0.874
T3 (High)	616	19 (3.1)	1.479 (0.616–3.709)	0.388

Adjusted factors for multivariate analyses included gender, age category, BMI category, disability of IADL, drinking status, smoking status, disease history, educational levels, time spent walking, and handgrip strength at baseline.

## Data Availability

Data cannot be shared for privacy or ethical reasons.

## Supplementary Materials

The following supporting information can be downloaded at: https://www.mdpi.com/article/10.3390/ijerph20227065/s1, Supplementary Figure S1. A Relationship between the rJDI12 scores and occurrence of muscle weakness after multiple imputations. Model 1 adjusted for gender, age category, and handgrip strength at baseline. Model 2 adjusted for model 1 and added the BMI category. Model 3 adjusted for model 2 and added disability of IADL, walking status, drinking status, smoking status, disease history, and educational status. Model 4 was adjusted for Model 3 and added energy intake. Model 5 adjusted for Model 3 and added protein intake. Continuous values were entered into each model for rJDI12. Multiple imputation was performed for all participants, with the exception of those who had missing baseline handgrip strength data.
