# Supplementary figures and images for: Association between Japanese Diet Adherence and Muscle Weakness in Japanese Adults Aged ≥50 Years: Findings from the JSTAR Cohort Study
Source: Int J Environ Res Public Health. 2023 Nov 15;20(22):7065. doi: 10.3390/ijerph20227065 (PMC10671671; doi:10.3390/ijerph20227065)

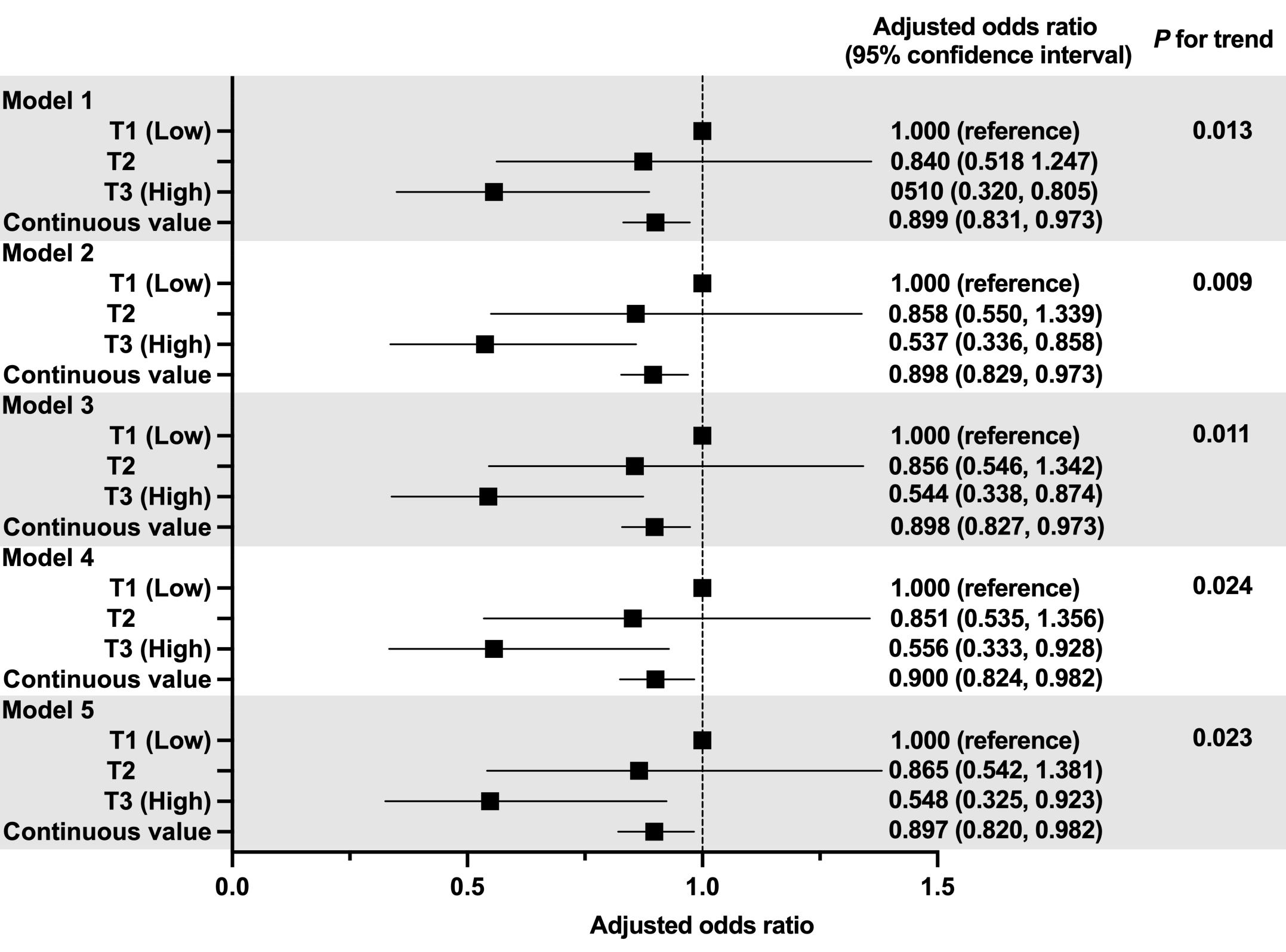

Supplement: Supplementary file 1 [file ijerph-20-07065-s001.zip › ijerph-2640810-supplementary.tiff]
